# Recovery of Fatty Acid Composition in Mediterranean Yellowtail (*Seriola dumerili*, Risso 1810) fed a Fish-Oil Finishing Diet

**DOI:** 10.3390/ijms21144871

**Published:** 2020-07-09

**Authors:** Francesco Bordignon, Silvia Martínez-Llorens, Angela Trocino, Miguel Jover-Cerdá, Ana Tomás-Vidal

**Affiliations:** 1 Department of Comparative Biomedicine and Food Science (BCA), University of Padova, Viale dell’Università 16, I-35020 Legnaro, Padova, Italy; francesco.bordignon.3@phd.unipd.it; 2 Institute of Animal Science and Technology, Group of Aquaculture and Biodiversity, Universitat Politècnica de València, Camino de Vera, 14, 46071 València, Spain; silmarll@dca.upv.es (S.M.-L.); mjover@dca.upv.es (M.J.-C.); atomasv@dca.upv.es (A.T.-V.)

**Keywords:** wash-out, greater amberjack, thrombogenicity, atherogenicity, EPA, DHA

## Abstract

The present study evaluated the effects of wash-out on the fatty acid (FA) composition in the muscles of Mediterranean yellowtail. After 109 days during which fish were fed either a fish oil (FO)-based diet (FO 100) or a diet (FO 0) in which FO was completely substituted by vegetable oils, all fish were subjected to a wash-out with FO 100 diet for 90 days. The FA profile of muscles in fish fed FO 0 diet at the beginning of the experiment reflected that of dietary vegetable oils, rich in linoleic acid (LA), and α-linolenic acid (ALA), and was deficient in AA (arachidonic acid), EPA (eicosapentaenoic acid), and DHA (docosahexaenoic acid). No essential FA were fully restored in fish previously fed FO 0 diet on 45th or 90th day of wash-out. At the end of wash-out, the FA composition showed that AA, EPA, and DHA in the white muscles increased by +33%, +16%, and +43% (*p* < 0.001), respectively. Similarly, AA and DHA in the red muscles increased by +33% and +41% respectively, while EPA remained similar to fish fed FO 0 diet exclusively. Therefore, a 90-d wash-out can partially improve the FA profile in muscles of Mediterranean yellowtail previously fed vegetable oil-based diets.

## 1. Introduction

Species diversification is considered as a tool for sustainable development of aquaculture in near future [1]. The Mediterranean yellowtail (*S. dumerili*, Risso 1810) is one of the most interesting candidates for European aquaculture diversification [2]. This carnivorous fish shows high growth rates (weighs 6 kg within 2.5 years of culture) and has excellent flesh quality and worldwide consumer acceptance [2,3].

Fish oil (FO) has been considered as the major lipid source in aquafeeds for carnivorous fish for a long time [4,5]. However, FO and fish meal production are no longer sustainable [6,7]. During the last couple of decades, the global supply of FO has been tightly regulated and has remained low and stable, whereas the aquaculture industry has expanded rapidly. Consequently, FO prices have increased, prompting researchers and industries to develop alternative lipid sources that can be included in aquafeeds [8].

Vegetable oils (VO) (e.g., soybean oil, linseed oil, palm oil, and rapeseed oil) have been widely tested as alternatives to FO in aquafeeds, specifically for their impact on fish growth and flesh quality [9,10,11]. However, VO are rich in C18 polyunsaturated fatty acids (PUFA), but are devoid of highly unsaturated fatty acids (HUFA), such as eicosapentaenoic acid (EPA; 20:5 n-3) and docosahexaenoic acid (DHA; 22:6 n-3), which are essentials for growth, health, reproduction, and body functions of fish [5]. Most freshwater fish are capable of desaturating and elongating C18 fatty acids (i.e., linoleic acid C18:2 n-6 and α-linolenic acid C18:3 n-3) into EPA and DHA [12], whereas marine carnivorous species have lost this bioconversion ability [5,13]. Therefore, these essential fatty acids (EFA) must be included in marine aquafeeds to meet their EFA requirement (approximately 4%) [12]. According to the literature, the partial substitution (up to 60%) of FO with VO in the diets of marine fish during the grow-out phase does not affect their mortality and growth rates [6,10].

Nevertheless, a major limitation of replacing dietary FO with VO is its effect on fillet composition. Since the fatty acid (FA) profile of fish tissues reflects those of the diets consumed by the fish [5,14], fillets of fish fed diets containing high VO levels may contain a low amount of n-3 HUFA [9,15], which are considered beneficial for human health [16,17,18,19]. High intakes of EPA and DHA are recommended to prevent premature birth and low birth weight [20], and also to reduce cardiovascular disease risks [21,22]. These FA are also anti-arrhythmic in nature and reduce platelet aggregation and blood triacyl glyceride levels [23]. For these reasons, health organizations of several countries recommend a daily intake of 1.2-2.0 g/d of n-3 HUFA [10], adjusted according to eating habits, age, and sex.

Therefore, to restore the healthy FA profile of fish fillets during fish farming, finishing strategies before slaughtering (i.e., wash-out period) have been proposed [5,15,24,25]. The efficacy of a finishing strategy including FO diets after a grow-out period supplemented with alternative lipid sources has been tested in marine fish species such as gilthead seabream [6,10], European seabass [11,13], red seabream [26], and Senegalese sole [27], as well as in freshwater species such as Murray cod [25,28], sunshine bass [29], and rainbow trout [30,31], and in Atlantic salmon [32,33,34,35].

Finishing strategy can also differently affect the FA composition of the red and the white muscles that make up the fish fillets [36]. The former (approx. 10% of the fillet) are located in strips along the midline and assure a steady aerobic swimming by an aerobic metabolism based on lipids [37,38]; the latter represent the bulk of the fillet [38] and use carbohydrates for their energy metabolism [38]. Additionally, white and red muscles show different sensorial traits and fatty acid composition [39].

To date, there is no information about the effects of a finishing/wash-out strategy in Mediterranean yellowtail with special emphasis on the time required to restore the FA profile and the sensitivity of selected FA to a wash-out diet. Thus, the present study aimed to evaluate the effects of a wash-out diet and the time required to restore the FA composition of the white and red muscles in Mediterranean yellowtail that was previously fed a vegetable oil-based diet.

## 2. Results

The feeding plan did not affect fish growth (Table 1). Fish reached an average weight of 490 g and 624 g after 45 and 90 d of wash-out, respectively, corresponding to SGR (specific growth rate) equal to 0.45% and 0.55% per day (Table 1).

At the beginning of the wash-out period, the FA profile in the white muscles of the fish fed FO 0 diet showed lower levels of saturated FA (SFA) (−6.1%), arachidonic acid (AA; C20:4 n-6) (−38%), EPA (C20:5 n-3) (−34%), DHA (C22:6 n-3) (−44%) but showed higher ALA (C18:3 n-3) (+176%; *p* < 0.001), and total n-6 (+11%) when compared to those seen in fish fed FO 100 diet. The higher total n-6 could be attributed to changes in the levels of LA (C18:2 n-6) (+16%) (Table 2). Similarly, the red muscles of the fish fed FO 0 diet showed lower levels of AA (−41%), EPA (−35%), and DHA (−47%), and higher ALA (+238%; *p* < 0.001) and total n-6 (+14%) (+21%), when compared to those seen in fish that were fed FO 100 diet (Table 2). The increase in total n-6 (+14%) could be attributed to the changes in LA.

The dietary treatment did not affect the average levels of C16:0 (14.47%), C18:0 (6.29%,), and total n-3 (21.39%) in the white muscles after 45 days of wash-out (Table 3). The FA profile of fish that were fed the finishing fish-oil diet (FO 0/FO 100) was similar to that of fish which were fed the VO diet exclusively. Fish that were previously fed FO 0 diet (groups FO 0/FO 0 and FO 0/FO 100) showed lower total SFA (−4%; *p* < 0.01), AA (−36%), DHA (−42%), but higher LA (+19%), total n-6 (+12%), and ALA (+193%) (*p* < 0.001) levels when compared with those seen in fish which were fed FO 100 diet exclusively (Table 3). On the other hand, the FA profile of the white muscles was partially restored as EPA showed an increase (+13%) when fish from FO 0/FO 0 dietary treatment were compared with fish that were fed FO 0/100 diet. The EPA levels of fish from FO 0/FO 0 dietary treatment were lower (−33%) than those seen in fish which were fed FO 100 diet exclusively (*p* < 0.001).

The dietary treatment did not affect the average levels of C16:0 (14.16%) and C18:0 proportions (6.78%) in the red muscles of the fish after 45 days of wash-out. Fish that were exclusively fed FO 0 diet and fish that were fed FO 0/FO 100 both showed lower total SFA (−4%) and DHA (−36%), but showed elevated proportions of LA (+18%) and total n-6 (+12%) (*p* < 0.001) when compared with those seen in fish fed FO 100 diet exclusively (Table 3). As seen in the white muscle, the FA profile of the red muscles was also partially restored as EPA showed an increase (+16%) when fish from FO 0/FO 0 dietary treatment were compared with fish that were fed FO 0/FO 100 diet. The EPA levels of fish from FO 0/FO 0 dietary treatment were lower (−21%) than those seen in fish which were always fed FO 100 diet (*p* < 0.001). Moreover, ALA decreased (−25%) in fish fed FO 0 diet exclusively, when compared with those seen in fish fed FO 0/FO 100 diets (*p* < 0.001) (Table 3).

At the end of wash-out phase, the dietary treatment did not affect the average levels of C16:0 (14.58%), C18:0 (6.13%), and total n-3 (20.91%) in the white muscles of the fish (Table 4). The differences recorded after 45 d of wash-out between fish that were exclusively fed FO 100 diet and those that were exclusively fed FO 0 diet or fed FO 0/FO 100 diets were confirmed for total SFA (+5% in FO 100; *p* < 0.001) and total *n*-6 (−6% in FO 100; *p* < 0.001). On the other hand, a partial restoration of the FA profile was observed for 74% of fatty acids in fish that were previously fed the FO 0 diet, due to the wash-out phase with FO 100 diet. Fish subjected to wash-out treatment showed higher ratios of AA (+33%), EPA (+16%), and DHA (+43%) (*p* < 0.001) and lower ratios of LA (−6%) and ALA (−29%) (*p* < 0.001) when compared with those seen in the fish that were not subjected to wash-out treatment (Table 4).

After 90 d of wash-out, the SFA and EPA ratios seen in the red muscles of fish that were exclusively fed FO 0 diet or in those which were subjected to wash-out treatment with FO 100 diet were lower than that seen in fish that were exclusively fed FO 100 diet [total SFA (−7%; *p* < 0.001) and EPA (−20%; *p* < 0.05)]. On the other hand, a partial restoration for 65% of fatty acids was observed in fish that were previously fed the FO 0 diet due to the wash-out treatment with FO 100 diet. Fish subjected to wash-out treatment showed higher ratios of AA (+33%) and DHA (+41%) (*p* < 0.001), but lower ratios of LA (−7%), and ALA (−29%) (*p* < 0.001) compared to fish exclusively fed with FO 0 diet (Table 4).

As for the nutritional quality of lipids, the index of atherogenicity (IA) was higher in white muscles of fish that were exclusively fed FO 100 diet when compared with those seen in the other groups of fish at the first (+23%), 45th (+17%), and 90th day of wash-out (+16%) (*p* < 0.001). No differences among treatments were recorded for the average value of the index of thrombogenicity (IT) at the first, 45th, and 90th day of wash-out treatment (0.23) (Table 5).

In the red muscles of the fish, IA was higher in fish that were exclusively fed FO 100 diet as compared to that in the fish which were fed FO 0 diet, on the first (+12%; *p* < 0.05) and on the 45th day of wash-out treatment (+16%; *p* < 0.001). In fish that were fed FO 0/FO 100 diet, IA value on the 45th day of wash-out (0.25, on average) was equal to that seen in fish that were fed FO 0 diet exclusively, whereas it was higher (+8%) at the end of the treatment. As for the white muscles, no differences among treatments were recorded for the average value of IT at the first, 45th, and 90th day of wash-out (0.22) (Table 5).

## 3. Discussion

In the last few decades, the widespread use of FO as lipid source in aquafeeds has drastically challenged the sustainability of aquaculture. The substitution of FO with alternative sources (such as vegetable oils) has been successful in terms of fish performance but has faced concerns about fish health and flesh nutritional quality. New feeding strategies are being developed to address those concerns. In fact, the replacement of FO with VO in aqua feed increases dietary oleic acid, LA and ALA and reduces the n-3 fatty acid such as EPA and DHA, which increases the vegetable lipid profile of the whole fish, as well as its organs and flesh [5]. Since n-3 PUFA play a specific role in inflammatory processes and immune response [40], the change in the dietary FA profile can affect fish response both during growth and wash-out. Moreover, the presence of vegetable FA leads to a decrease in the nutritional value of fish flesh for humans as a result of changes in FA profile as well as an unbalanced ratio of n-3/n-6 [41]. This can affect both the white and red muscles of fish, whereas a greater contribution to the nutritional value of the flesh comes from the former due its higher proportion on the whole fillet compared to the latter (in the present trial, red muscles: 6.8–9.8% of total fillet weight).

In the present trial, two indices based on the functional effects of FA were used to evaluate the nutritional quality of the fish fillet lipid fraction, i.e., the indexes of atherogenicity (IA) and thrombogenicity (IT). Briefly, IA indicates the relationship between main classes of saturated FA (considered pro-atherogenic, i.e., favoring lipid adhesion to cells) and those of unsaturated FA (anti-atherogenic; i.e., inhibiting the aggregation of lipid plaque and reducing the levels of cholesterol, phospholipid and esterified FA) [42]. The IT is defined by the relationship between the pro-thrombogenic saturated FA and the anti-thrombogenic FA (MUFA, PUFA n-6, and PUFA n-3) [42]. The IT of flesh in our trial was consistent with values (0.22–0.23) found in most common marine species [43], whereas the higher IA in fish fed FO 100 diet is related to the higher content of myristic acid (C14:0) in their red and white muscles. In fact, myristic acid in FO 100 diet was almost twice than in FO 0 one. Nevertheless, for all fish, IA values were within recommended values for human health (<1.0) [44,45].

Moreover, in the present trial, after the growth phase, the FA profile of *S. dumerili* in the white and red muscles reflected the FA composition of the fish-oil (FO 100) or vegetable-oil (FO 0) diets, ingested by the fish, which agreed with our assumptions. Nevertheless, the differences in AA, EPA and DHA rates in both the white and red muscles of the fish that were fed the two dietary treatments were lower than the difference in the diets, which agrees with previously published studies on gilthead seabream [6,10,46] and rainbow trout [31].

To restore the fillet nutritional value (in terms of high levels of EPA and DHA) in fish grown on diets containing vegetable oils, specific feeding strategies can be used during the finishing period to wash-out fish that were previously fed VO diets [4,15,31]. However, fish FA levels are also affected by the fish biosynthesis ability for the different FA, besides the dietary supply. Indeed, standards for a successful wash-out and recovery of the desired FA in fish flesh are not yet available for all species including greater amberjack. On the other hand, the available literature about other species is inconsistent.

In gilthead seabream, oleic acid and LA are retained in the flesh of the fish even after 120 d of wash-out [6]. In European sea bass, Montero et al. [11] reported that LA was 3-fold higher in fish previously fed vegetable-oil diets compared to those that were fed on fish oil exclusively even after 150 d of wash-out, which agrees with the results stated by Izquierdo et al. [10] for seabass after a 104-d wash-out. In turbot, even after a wash-out of eight months, high levels of LA in muscle phospholipids have been reported, which could be attributed to the poor LA utilization in a species that can convert LA to 20:2 n-6 [47].

According to Mourente and Bell [13] the wash-out (150 d, 160 g LW) treatment in sea bass is insufficient for restoring EFA. On the contrary, Montero et al. [11] report that a wash-out period of 150 d, after 8 months of a diet containing 60:40 ratio of vegetable oil and fish oil (75–366 g LW), was able to recover flesh DHA, but could not increase EPA level. Similarly, in gilthead seabream, EFA levels could not be restored after a 120-d wash-out [6]. On the other hand, Izquierdo et al. [10] report that DHA and AA levels were recovered in gilthead seabream following a wash-out of 60 d (after 7 months of feeding with diets containing vegetable oils at 60% and 80%; 85–452 g LW), but EPA levels were not recovered even after 90 d of wash-out treatment.

In rainbow trout, 8–12 weeks of wash-out cannot recover EPA and DHA levels [30,31], whereas in Atlantic salmon that has been previously fed linseed oil-based diets for 40 weeks, levels of EPA and DHA in flesh can be restored by 80% after 20 weeks [34] and by 83% after 24 weeks of wash-out treatment [32]. On the other hand, Bell et al. [33] report that in post-smolt salmon (200 g) previously fed a diet containing increasing rates of rapeseed oil for 16 weeks, EPA and DHA levels in flesh can be recovered after 4 and 12 weeks of wash-out, respectively. In Senegalese sole a total restoration of all EFA levels in flesh can be achieved after 26 d of wash-out [27].

In the present study, EFA levels in the Mediterranean yellowtail did not recover completely after a 90-d wash-out, but some differences between white and red muscles were recorded. In the white muscles, AA, EPA, and DHA levels were partially restored, despite remaining lower when compared with those of fish fed fish-oil diets exclusively. On the other hand, in the red muscle, a partial restoration was observed only for AA and DHA levels, whereas the EPA level remained low.

Based on literature, EFA recovery in fish muscles depends upon several factor such as fish species, fish size, duration for which fish were fed vegetable-oil based diets, duration of the wash-out period, and the specific FA. Additionally, FA incorporation in fish muscles can be altered by different metabolic factors such as FA elongation and desaturation, β-oxidation [48], preferential incorporation [49], lipogenic activity, environmental factors [50], size and age of animals [51], and their physiological state [52].

In the present trial, after 90 d of wash-out, DHA was partially recovered and selectively retained by the muscles. This observation agrees with previously published studies on a wide variety of species [53]. The mechanism of selective deposition has been likely influenced by the high specificity of fatty acid transferases for DHA and the relative resistance of DHA to β-oxidation [54]. Fish size and fish physiological state can also play a role in DHA recovery. DHA content in flesh of the fish has been found to be negatively affected by the increase in fish size [55] and the competition between muscles and developing gonads for its incorporation at the time of sexual maturation [52], which was not the case of our trial.

The recovery of EPA in the flesh of the fish following a wash-out strategy was unsuccessful in the present trial. Our observations agree with previously reported studies done with other species (seabass, [11]; seabream, [10]; rainbow trout, [30,31]). According to Madsen et al. [56], a preferential oxidation of EPA occurs over DHA; and EPA is mainly oxidized by mitochondria, whereas DHA seems to be oxidized by the peroxisomes. Thus, the failure of EPA recovery in the white muscle of Mediterranean yellowtail in our trial could be attributed to the fact that mitochondrial β-oxidation prevails over peroxisomal oxidation in white muscles [56].

The definition of the wash-out duration is also crucial to produce fish with a healthy FA profile. An n-3 HUFA deficiency in the fish muscle lowers the nutritional value of the fish for humans. To overcome this, a dilution model has been proposed [15] to predict the FA restoration at a given time after a dietary change. This model has been used in some fish species such as gilthead seabream and Atlantic salmon [15,57]. Nevertheless, the model does not fully represent changes in all FA and the variation in different species. For instance, in the Murray cod, the mobilization of oleic acid, LA, and ALA during the wash-out has been found to be at a lower rate than the rate predicted by the model, with major changes occurring during the first days of wash-out [25]. According to some studies [15,29], the model can provide misleading results when it is used to study the FA changes of ‘lean’ fish fillets, such as the Mediterranean yellowtail.

Thus, under the conditions of our trial, a wash-out period of 90 d partially improved the final FA profile in muscles of Mediterranean yellowtail that was previously fed VO-based diets. In fact, based on our results, the quantity of muscle necessary to cover the daily-recommended ingestion of EPA and DHA (average requirement of 1.6 g/d of n-3 HUFA) decreased from 90 g/d (fish submitted to feeding plan FO 0/FO 0) to 70 g/d (fish submitted to feeding plan FO 0/FO 100). However, further studies are necessary to define the time and the dietary fish oil level that can restore EFA to the same levels seen in the fish that were exclusively fed on fish oil for *S. dumerili*, under different conditions of growth and at different sizes.

## 4. Materials and Methods

### 4.1. Experimental Diets

Two isoproteic (59% crude protein, 50% digestible protein) and isolipidic (15% crude lipid) extruded diets were formulated: a control diet (FO 100) with fish oil as a unique lipid source and a diet (FO 0) in which FO was completely substituted by a blend of vegetable oils (linseed oil, sunflower oil, and palm oil in the ratio of 4:3:3). Diets were prepared using a cooking-extrusion process with a semi-industrial twin-screw extruder (CLEXTRAL BC-45; Firmity, St Etienne, France), at 100 rpm speed screw, 110 °C temperature, and at 40–50 atm pressure to obtain pellets with 2–3 mm diameter. Ingredients and chemical composition of the experimental diets are presented in Table 6 and their fatty acid composition is given in Table 7.

### 4.2. In Vivo Trial

The trial was performed at the Laboratory of Aquaculture (LAC) of the Department of Animal Science at the Polytechnic University of Valencia (Valencia, Spain) in accordance with the protocol approved (15/04/2015) by the Committee of Ethics and Animal Welfare of the Universitat Politècnica de València (UPV) and following the Spanish Royal Decree 53/2013 about the protection of animals used for scientific purposes. The facility presented a thermo-regulated recirculation seawater system (65 m^3^ capacity), with a rotary drum type filter and a mechanical gravity biofilter of 2 m^3^, equipped with aerated cylindrical fiberglass tanks of 1750 L capacity. Water temperature during the trial was 21.5 ± 2.4 °C.

**Figure 1:**
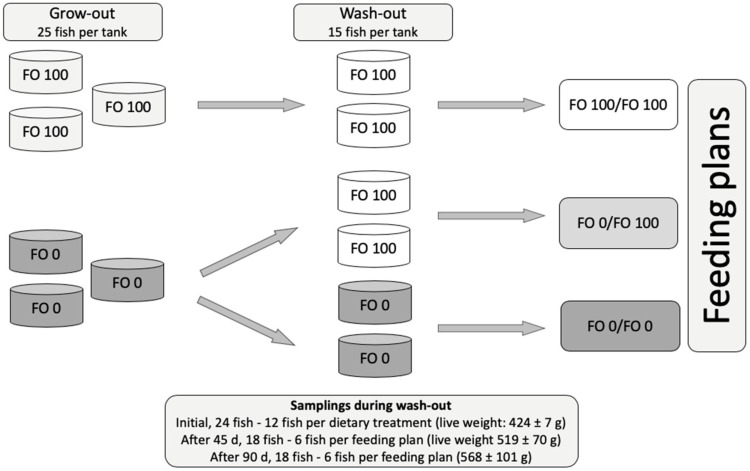
Scheme of feeding plans and samplings. During grow-out (109 d), fish were fed FO 100 diets (formulated with fish oil as lipid source) or FO 0 diets (formulated with vegetable oil as lipid source) (three tanks per experimental diet). At the end of grow-out, the same fish were moved into 6 tanks and fed FO 100 diets or FO 0 diets for the wash-out period (90 d) resulting into three feeding plans: FO 0/FO 0, FO 0/FO 100, and FO 100/FO 100 (two tanks per feeding plan). Fish were sampled at the beginning, and after 45 d and 90 d of wash-out.

Yellowtails of both sexes, 11 months of age, used in this trial underwent a grow-out period in which they were fed FO 100, FO 25 (75% vegetal oil) and FO 0 diets (three tanks/treatment; 25 fish/tank) for 109 d [58]. Then, the fish that were fed FO 0 and FO 100 diets were moved into 6 tanks and were fed FO 0 diets as the grow-out or FO 100 diets for the next 90 d (wash-out period), as per the following feeding plans: FO 0/FO 0, FO 0/FO 100, and FO 100/FO 100 (2 tanks per experimental group; 15 fish per tank) (Figure 1). Fish that were previously fed FO 25 diet were not considered for the present study.

Feed was offered by hand, twice a day (at 09.00 and 16.00), six days a week, until apparent visual satiation. Fish were weighed at the beginning, at 45 d and at 90 d of wash-out. Fish weight was used to calculate the specific growth rate.

At the beginning of the wash-out phase, 12 fish per treatment were euthanized with a lethal bath of clove oil (150 mg·L^−1^) and were dissected for fillet sampling. During the wash-out period, 6 other fish per treatment were euthanized and dissected with the same procedure after 45 d and after 90 d (Figure 1). At each sampling (beginning, 45 d and 90 d of wash-out), the right whole fillets (without skin and bone) were excised, vacuum packed, and stored at −80 °C until analyses.

### 4.3. Chemical Analysis

Experimental diets were analysed following AOAC procedures [59]: dry matter (incinerated at 105 °C to constant weight), ash (incinerated at 550 °C to constant weight), and crude lipid of diets and fish muscles were extracted with diethyl ether (ANKOM^XT10^; ANKOM Technology, Macedon, NY, USA). Crude protein was determined following the Dumas combustion method, using a LECO CN628 apparatus (LECO, St Joseph, MI, USA). Gross energy content (GE) was calculated according to Brouwer [60], from the C (g) and N (g) balance in the feed (GE = 51.8 × C − 19.4 × N). All analyses were performed in triplicate. Fatty acids were analysed twice on the same single sample.

For each fish, the white and red muscles of each fillet were separately ground, freeze-dried, and ground again before their chemical analyses. Fatty acid methyl esters (FAMEs) were obtained directly from freeze-dried samples [61]. One milliliter of tridecanoic acid (C13:0) was used as an internal standard. Then, 0.7 mL of 10 N KOH and 5.3 mL of HPLC grade methanol were added to the tubes. Tubes were incubated at 55 °C in a thermoblock for 1.5 h, and underwent vigorous shaking for 5 s at an interval of 20 min. The tubes were cooled down to ambient temperature in a water bath, and 1.5 mL of HPLC grade hexane was added to the reaction tubes, which were then vortexed and centrifuged at 1006× *g* for 5 min. In the next step, the hexane layer containing the FAMEs, was transferred into separate vials for gas chromatography. The vials were kept at −80 °C until they were analysed by gas chromatography. The FAMEs were analysed in a Focus Gas Chromatograph (Thermo, Milan, Italy) equipped with a split/splitless injector and a flame ionisation detector. Separation of the methyl esters was performed in a SPTM 2560 fused silica capillary column (Supelco, Bellafonte, PA, USA) (film thickness: 100 m × 0.25 mm × 0.2 μm). Helium was used as the carrier gas, at a flow rate of 20 cm s^−1^. Samples were injected with a split ratio of 1:100.

The initial oven temperature set at 140 °C, was kept constant for 5 min, and then increased to 240 °C at the rate of 4 °C·min^−1^, which was then maintained for 30 min. The FA were identified by comparing their retention times with those of the standards supplied by Supelco (Sigma-Aldrich, St. Louis, Missouri, USA). The content of each FA was expressed as the percentage of the total FA content.

### 4.4. Nutritional Indexes

The index of atherogenicity (IA) and the index of thrombogenicity (IT) were calculated according to Ulbricht and Southgate [62] as stated below, where MUFA are monounsaturated fatty acids:

(1) $$\mathrm{IA}=\frac{C12:0+\left(4*C14:0\right)+C16:0}{\mathrm{Total}\;\mathrm{MUFA}+n6\;\mathrm{PUFA}+\;n3\;\mathrm{PUFA}}$$

(2) $$\;\mathrm{IT}\;=\frac{C14:0+C16:0+C18:0}{\left(0.5*\mathrm{Total}\;\mathrm{MUFA}+0.5*n6\;\mathrm{PUFA}+3*n3\;\mathrm{PUFA}\right)+\frac{n3\;\mathrm{PUFA}}{n6\;\mathrm{PUFA}}}\;$$

### 4.5. Statistical Analysis

The data of growth performance, muscle lipid content, FA composition, and nutritional indexes at the start of the wash-out phase were analysed by analysis of variance with the diet fed during grow-out phase (FO 0, FO 100) as the main effect. The same data of fillets collected on the 45th and 90th day of wash-out were analysed by ANOVA with the feeding plan (FO 0/FO 0, FO 0/FO 100, FO 100/FO 100) as the main effect. The PROC GLM of the Statistical Analysis System (version 9.3, SAS Institute, Cary, NC, USA) [63] was used for all analyses. Adjusted means were compared by Bonferroni *t*-test. Differences between means with *p* ≤ 0.05 were accepted as statistically significant differences. The data of FA composition were preliminarily tested for a normal distribution using the Shapiro–Wilk statistic and the PROC UNIVARIATE [63]. The data of FA per mg of tissue were analysed as described for FA composition and are available as supplementary materials (Tables S1–S3).

## Figures and Tables

**Table 1:** Growth performance of *S. dumerili* during the wash-out period. Values are expressed as least square (LS) means.

	Feeding Plan			*p*-Value	RSD
	FO 100/FO 100	FO 0/FO 0	FO 0/FO 100		
Live weight (g)					
Initial	401	397	397	0.223	5.39
45 d	494	486	491	0.947	49.8
90 d	629	629	612	0.798	62.5
Specific growth rate, %/d					
0–45 d	0.46	0.42	0.46	0.904	0.23
45–90 d	0.53	0.61	0.50	0.706	0.29
0–90 d	0.49	0.51	0.48	0.861	0.11

FO 100: diet formulated with fish oil as lipid source. FO 0: diet in which fish oil was totally substituted by vegetable oils. The feeding plan gives the diet fed during grow-out/the diet fed during wash-out. RSD: residual standard deviation.

**Table 2:** Fat content (%) and fatty acid composition (% of total fatty acids content) of white and red muscles at the beginning of the wash-out period in *S. dumerili* always fed FO 100 and FO 0 diets (12 fish per diet): effect of the grow-out diet. Values are expressed as least square (LS) means.

	White Muscle				Red Muscle			
	FO 100	FO 0	*p*-Value	RSD	FO 100	FO 0	*p*-Value	RSD
Fat, %	5.95	6.04	0.803	0.791	4.33	4.47	0.74	0.928
Fatty acids, %								
14:0	2.27	1.47	<0.001	0.141	1.82	1.26	<0.01	0.358
15:0	Tr	Tr			0.15	0.03	0.02	0.104
16:0	14.96	14.22	<0.01	0.437	14.50	14.40	0.70	0.558
17:0	0.40	0.23	<0.001	0.035	0.44	0.24	<0.001	0.042
18:0	5.89	6.12	0.02	0.197	6.53	6.83	<0.01	0.181
20:0	0.36	0.32	<0.001	0.011	0.38	0.32	<0.001	0.020
22:0	0.12	0.17	0.03	0.039	0.12	0.16	<0.001	0.016
∑ SFA ^1^	24.06	22.59	<0.001	0.639	24.01	23.34	0.10	0.873
14:1 n-9	0.28	0.15	<0.001	0.023	0.11	0.10	0.96	0.109
16:1 n-9	3.78	2.36	<0.001	0.210	3.27	2.06	<0.001	0.361
17:1 n-10	0.33	0.17	<0.001	0.025	0.32	0.16	<0.001	0.026
18:1 n-7	4.12	3.20	<0.001	0.179	4.22	3.27	<0.001	0.278
18:1 n-9	27.55	31.72	<0.001	0.821	27.00	31.70	<0.001	0.799
20:1 n-9	1.72	0.76	<0.001	0.168	1.91	0.74	<0.001	0.169
22:1 n-9	0.28	0.12	<0.001	0.046	0.36	0.13	<0.001	0.112
24:1 n-9	0.34	0.19	<0.001	0.047	0.41	0.20	<0.001	0.046
∑ MUFA	38.40	38.67	0.46	0.712	37.60	38.36	0.05	0.771
18:2 n-6	14.20	16.45	<0.001	0.541	13.01	15.77	<0.001	0.522
18:3 n-6	0.14	0.13	<0.01	0.009	0.13	0.10	<0.01	0.017
20:3 n-6	0.10	0.05	<0.001	0.012	0.11	0.07	0.07	0.040
20:4 n-6	0.77	0.48	<0.001	0.071	0.92	0.54	<0.001	0.059
22:4 n-6	0.39	0.18	<0.001	0.050	0.51	0.21	<0.001	0.046
∑ n-6 PUFA	15.60	17.29	<0.001	0.466	14.68	16.69	<0.001	0.460
18:3n-3	4.08	10.84	<0.001	1.177	2.90	9.81	<0.001	0.948
20:3n-3	0.20	0.25	<0.01	0.022	0.20	0.27	<0.001	0.029
20:5n-3	3.44	2.26	<0.001	0.209	3.27	2.14	<0.001	0.156
22:5n-3	1.68	1.05	<0.001	0.143	2.12	1.29	<0.001	0.183
22:6n-3	11.28	6.37	<0.001	1.319	14.02	7.49	<0.001	1.063
∑ n-3 PUFA	20.68	20.77	0.86	1.159	22.51	21.00	<0.01	0.972
20:2	0.85	0.49	<0.001	0.056	0.80	0.45	<0.001	0.067
22:2	0.41	0.19	<0.001	0.037	0.40	0.16	<0.001	0.037
∑ PUFA	37.53	38.74	0.03	1.093	38.39	38.30	0.98	0.892
DHA/EPA	3.27	2.81	<0.01	0.258	4.30	3.50	<0.001	0.344

SFA: saturated fatty acids; MUFA: monounsaturated fatty acids; PUFA: polyunsaturated fatty acids; RSD: residual standard deviation; DHA/EPA: C22:6 n-3/C20:5 n-3. FO 100: diet formulated with fish oil as lipid source. FO 0: diet in which fish oil was totally substituted by vegetable oils. ^1^ Total SFA include fatty acids not listed (<0.1% of total FA), C6:0, C8:0, C10:0, C11:0, C12:0, C13:0, C21:0, C23:0, C24:0.

**Table 3:** Fat content (%) and fatty acid composition (% of total fatty acid content) of white and red muscle in *S. dumerili* fed FO 100/FO 100, FO 0/FO 0, and FO 0/FO 100 diets after 45 d of wash-out (6 fish per feeding plan): effect of the feeding plan. Values are expressed as least square (LS) means.

	White Muscle					Red Muscle				
Feeding Plan	FO 100/FO 100	FO 0/FO 0	FO 0/FO 100	*p*-Value	RSD	FO 100/FO 100	FO 0/FO 0	FO 0/FO 100	*p*-Value	RSD
Fat, %	4.79	4.65	5.42	0.554	1.057	4.37	4.67	4.42	0.842	0.808
Fatty acids, %										
14:0	2.11 ^b^	1.41 ^a^	1.60 ^a^	<0.001	0.103	1.80 ^b^	1.21 ^a^	1.33 ^a^	<0.001	0.087
15:0	0.27 ^c^	0.14 ^a^	0.17 ^b^	<0.001	0.014	0.25 ^c^	0.12 ^a^	0.16 ^b^	<0.001	0.011
16:0	14.68	14.36	14.36	0.42	0.431	14.39	14.22	13.88	0.17	0.385
17:0	0.39 ^c^	0.22 ^a^	0.26 ^b^	<0.001	0.020	0.40 ^c^	0.22 ^a^	0.27 ^b^	<0.001	0.017
18:0	6.20	6.38	6.29	0.50	0.223	6.71	6.82	6.82	0.64	0.224
20:0	0.36 ^b^	0.30 ^a^	0.32 ^a^	<0.001	0.018	0.37 ^b^	0.30 ^a^	0.32 ^a^	<0.001	0.017
22:0	0.12 ^a^	0.16 ^b^	0.15 ^b^	<0.001	0.010	0.12 ^a^	0.17 ^b^	0.14 ^b^	<0.01	0.013
∑ SFA ^1^	24.19 ^b^	23.03 ^a^	23.24 ^a^	<0.01	0.427	24.12 ^b^	23.15 ^a^	23.03 ^a^	<0.01	0.496
16:1 n-9	3.60 ^c^	2.28 ^a^	2.61 ^b^	<0.001	0.134	3.19 ^c^	2.03 ^a^	2.29 ^b^	<0.001	0.112
17:1 n-10	0.34 ^b^	0.17 ^a^	0.21 ^a^	<0.001	0.020	0.33 ^c^	0.17 ^a^	0.21 ^b^	<0.001	0.020
18:1 n-7	4.20 ^c^	3.09 ^a^	3.39 ^b^	<0.001	0.145	4.41 ^c^	3.37 ^a^	3.68 ^b^	<0.001	0.119
18:1 n-9	26.97 ^a^	32.09 ^b^	30.69 ^b^	<0.001	0.886	26.83 ^a^	32.10 ^c^	30.38 ^b^	<0.001	0.854
20:1 n-9	1.80 ^c^	0.72 ^a^	0.98 ^b^	<0.001	0.129	1.85 ^c^	0.75 ^a^	1.10 ^b^	<0.001	0.124
22:1 n-9	0.31 ^b^	0.11 ^a^	0.15 ^a^	<0.001	0.041	0.35 ^c^	0.14 ^a^	0.21 ^b^	<0.001	0.029
24:1 n-9	0.41 ^b^	0.19 ^a^	0.22 ^a^	<0.001	0.038	0.41 ^b^	0.21 ^a^	0.26 ^a^	<0.001	0.033
∑ MUFA	37.63	38.65	38.25	0.15	0.769	37.37 ^a^	38.77 ^b^	38.13 ^a,b^	0.02	0.644
18:2 n-6	13.19 ^a^	15.92 ^b^	15.44 ^b^	<0.001	0.436	12.50 ^a^	15.16 ^b^	14.33 ^b^	<0.001	0.489
18:3 n-6	0.14 ^b^	0.13 ^a^	0.13 ^a^	<0.001	0.006	0.12	0.11	0.11	0.45	0.010
20:3 n-6	0.10 ^b^	0.05 ^a^	0.06 ^a^	<0.001	0.013	0.11 ^b^	0.06 ^a^	0.07 ^a^	0.001	0.017
20:4 n-6	0.91 ^b^	0.54 ^a^	0.61 ^a^	<0.001	0.084	0.95 ^b^	0.59 ^a^	0.74 ^a,b^	<0.001	0.073
22:4 n-6	0.48 ^b^	0.18 ^a^	0.25 ^a^	<0.001	0.045	0.53 ^c^	0.22 ^a^	0.32 ^b^	<0.001	0.045
∑ n-6 PUFA	14.82 ^a^	16.82 ^b^	16.49 ^b^	<0.001	0.402	14.21 ^a^	16.14 ^b^	15.57 ^b^	<0.001	0.406
18:3 n-3	3.27 ^a^	10.30 ^b^	8.83 ^b^	<0.001	0.945	2.91 ^a^	9.21 ^c^	7.35 ^b^	<0.001	0.813
20:3 n-3	0.20 ^a^	0.26 ^b^	0.25 ^b^	<0.01	0.026	0.20 ^a^	0.29 ^b^	0.30 ^b^	<0.001	0.033
20:5 n-3	3.47 ^c^	2.18 ^a^	2.47 ^b^	<0.001	0.126	3.13 ^c^	2.13 ^a^	2.48 ^b^	<0.001	0.126
22:5 n-3	1.84 ^b^	1.10 ^a^	1.25 ^a^	<0.001	0.153	2.21 ^b^	1.48 ^a^	1.72 ^a^	<0.001	0.154
22:6n-3	13.32 ^b^	7.00 ^a^	8.44 ^a^	<0.001	1.296	14.67 ^b^	8.19 ^a^	10.63 ^a^	<0.001	1.293
∑ n-3 PUFA	22.10	20.84	21.24	0.06	0.741	23.12 ^b^	21.30 ^a^	22.48 ^a,b^	0.04	0.945
20:2	0.84 ^b^	0.48 ^a^	0.55 ^a^	<0.001	0.051	0.79 ^c^	0.47 ^a^	0.56 ^b^	<0.001	0.045
22:2	0.42 ^b^	0.18 ^a^	0.23 ^a^	<0.001	0.036	0.39 ^c^	0.17 ^a^	0.23 ^b^	<0.001	0.027
∑ PUFA	38.17	38.31	38.52	0.79	0.772	38.51	38.08	38.84	0.39	0.763
DHA/EPA	3.83	3.23	3.42	0.13	0.438	4.69 ^b^	3.86 ^a^	4.28 ^a,b^	0.04	0.428

SFA: saturated fatty acids, MUFA: monounsaturated fatty acids, PUFA: polyunsaturated fatty acids, RSD: residual standard deviation, DHA/EPA: C22:6 n-3/C20:5 n-3. FO 100: diet formulated with fish oil as lipid source. FO 0: diet in which fish oil was totally substituted by vegetable oils. The feeding plan gives the diet fed during grow-out/the diet fed during wash-out. ^a,b,c^ Means with different superscript letter statistically differ. ^1^ Total SFA include fatty acids not listed (<0.1% of total FA), C6:0, C8:0, C10:0, C11:0, C12:0, C13:0, C21:0, C23:0, C24:0.

**Table 4:** Fat content (%) and fatty acid composition (% of total fatty acid content) of white and red muscle in *S. dumerili* fed FO 100/FO 100, FO 0/FO 0, and FO 0/FO 100 diets after 90 d of wash-out (6 fish per feeding plan): effect of the feeding plan. Values are expressed as least square (LS) means.

	White Muscle					Red Muscle				
Feeding Plan	FO 100/FO 100	FO 0/FO 0	FO 0/FO 100	*p*-Value	RSD	FO 100/FO 100	FO 0/FO 0	FO 0/FO 100	*p*-Value	RSD
Fat, %	5.67	4.68	5.21	0.51	1.455	5.22 ^b^	3.65 ^a^	4.99 ^b^	<0.01	0.986
Fatty acids, %										
14:0	2.24 ^c^	1.46 ^a^	1.74 ^b^	<0.001	0.110	1.88 ^b^	1.22 ^a^	1.51 ^a^	<0.001	0.143
15:0	0.29 ^c^	0.14 ^a^	0.20 ^b^	<0.001	0.012	0.27	0.13	0.39	0.07	0.176
16:0	14.79	14.41	14.53	0.27	0.393	14.74 ^b^	14.04 ^a^	14.17 ^a^	0.03	0.446
17:0	0.41 ^c^	0.23 ^a^	0.30 ^b^	<0.001	0.015	0.42 ^c^	0.23 ^a^	0.31 ^b^	<0.001	0.017
18:0	6.15	6.18	6.07	0.80	0.258	6.92 ^b^	6.69 ^b^	6.42 ^a^	0.01	0.226
20:0	0.36 ^c^	0.29 ^a^	0.33 ^b^	<0.001	0.021	0.37 ^b^	0.32 ^a^	0.32 ^a^	<0.001	0.016
22:0	0.11 ^a^	0.15 ^b^	0.14 ^b^	<0.001	0.012	0.12 ^a^	0.17 ^c^	0.14 ^b^	<0.001	0.010
∑ SFA ^1^	24.41 ^b^	22.94 ^a^	23.38 ^a^	<0.001	0.519	24.83 ^b^	22.90 ^a^	23.37 ^a^	<0.001	0.612
16:1 n-9	3.76 ^c^	2.34 ^a^	2.85 ^b^	<0.001	0.144	3.21 ^c^	2.04 ^a^	2.56 ^b^	<0.001	0.204
17:1 n-10	0.33 ^c^	0.16 ^a^	0.23 ^b^	<0.001	0.023	0.34 ^c^	0.17 ^a^	0.24 ^b^	<0.001	0.025
18:1 n-7	4.15 ^c^	3.00 ^a^	3.69 ^b^	<0.001	0.363	4.32 ^c^	3.30 ^a^	3.80 ^b^	<0.001	0.133
18:1 n-9	27.13 ^a^	33.07 ^c^	30.97 ^b^	<0.001	0.685	26.41 ^a^	32.84 ^c^	30.39 ^b^	<0.001	0.819
20:1 n-9	1.84 ^c^	0.70 ^a^	1.16 ^b^	<0.001	0.079	1.90 ^c^	0.75 ^a^	1.25 ^b^	<0.001	0.076
22:1 n-9	0.33 ^c^	0.09 ^a^	0.20 ^b^	<0.001	0.029	0.38 ^c^	0.14 ^a^	0.23 ^b^	<0.001	0.011
24:1 n-9	0.42 ^c^	0.18 ^a^	0.26 ^b^	<0.001	0.039	0.51 ^b^	0.23 ^a^	0.30 ^a^	<0.001	0.043
∑ MUFA	37.96 ^a^	39.54 ^b^	39.36 ^b^	0.02	0.883	37.07 ^a^	39.47 ^b^	38.77 ^a^	<0.01	1.114
18:2 n-6	13.35 ^a^	15.39 ^c^	14.51 ^b^	<0.001	0.384	12.41 ^a^	14.84 ^c^	13.81 ^b^	<0.001	0.361
18:3 n-6	0.12	0.12	0.11	0.80	0.024	0.12	0.11	0.14	0.43	0.040
20:3 n-6	0.19	0.07	0.13	0.21	0.110	0.11 ^c^	0.07 ^a^	0.08 ^b^	<0.001	0.004
20:4 n-6	0.92 ^c^	0.48 ^a^	0.64 ^b^	<0.001	0.056	1.02 ^c^	0.54 ^a^	0.72 ^b^	<0.001	0.095
22:4 n-6	0.38 ^b^	0.20 ^a^	0.27 ^a,b^	<0.001	0.061	0.50 ^b^	0.21 ^a^	0.31 ^a^	<0.001	0.092
∑ n-6 PUFA	14.96 ^a^	16.26 ^b^	15.66 ^b^	<0.001	0.405	14.16 ^a^	15.74 ^c^	15.06 ^b^	<0.001	0.327
18:3 n-3	2.70 ^a^	10.84 ^c^	7.73 ^b^	<0.001	0.271	2.26 ^a^	9.56 ^c^	6.82 ^b^	<0.001	0.208
20:3 n-3	0.18 ^a^	0.28 ^b^	0.25 ^b^	<0.001	0.026	0.18 ^a^	0.33 ^c^	0.26 ^b^	<0.001	0.030
20:5 n-3	3.44 ^c^	2.23 ^a^	2.58 ^b^	<0.001	0.150	3.00 ^b^	2.31 ^a^	2.48 ^a^	0.02	0.396
22:5 n-3	1.86 ^c^	1.04 ^a^	1.28 ^b^	<0.001	0.052	2.14 ^c^	1.41 ^a^	1.64 ^b^	<0.001	0.064
22:6 n-3	13.24 ^c^	6.20 ^a^	8.87 ^b^	<0.001	0.909	15.23 ^c^	7.61 ^a^	10.71 ^b^	<0.001	1.453
∑ n-3 PUFA	21.42	20.59	20.71	0.28	0.897	22.81	21.22	21.91	0.13	1.362
20:2	0.83 ^c^	0.49 ^a^	0.62 ^b^	<0.001	0.026	0.75 ^c^	0.49 ^a^	0.60 ^b^	<0.001	0.054
22:2	0.42 ^c^	0.18 ^a^	0.27 ^b^	<0.001	0.022	0.38 ^c^	0.18 ^a^	0.29 ^b^	<0.001	0.042
∑ PUFA	37.63	37.52	37.26	0.71	0.697	38.10	37.63	37.86	0.81	1.302
DHA/EPA	3.86 ^b^	2.78 ^a^	3.44 ^b^	<0.001	0.297	5.10 ^b^	3.41 ^a^	4.33 ^a,b^	<0.01	0.714

SFA saturated fatty acids, MUFA monounsaturated fatty acids, PUFA polyunsaturated fatty acids, RSD residual standard deviation, DHA/EPA: C22:6 n-3/C20:5 n-3. FO 100: diet formulated with fish oil as lipid source. FO 0: diet in which fish oil was totally substituted by vegetable oils. The feeding plan gives the diet fed during grow-out/the diet fed during wash-out. ^a,b,c^ Means with different superscript letter statistically differ. ^1^ Total SFA include fatty acids not listed (<0.1% of total FA), C6:0, C8:0, C10:0, C11:0, C12:0, C13:0, C21:0, C23:0, C24:0.

**Table 5:** Indices of nutritional quality of lipids and EPA + DHA content (g/100 g of muscle) of white and red muscles in *S. dumerili* at the beginning of the wash-out period (0 d) and after 45 and 90 d: effect of the feeding plan. Values are expressed as least square (LS) means.

	Feeding Plan			*p*-Value	RSD
	FO 100/FO 100	FO 0/FO 0	FO 0/FO 100		
	White muscle				
Initial sampling					
Index of atherogenicity	0.32	0.26	-	<0.001	0.014
Index of thrombogenicity	0.24	0.23	-	0.062	0.015
PUFA n-6/PUFA n-3	0.76	0.83	-	0.008	0.053
EPA + DHA	2.57	1.73	-	<0.001	0.322
Sampling at 45 d					
Index of atherogenicity	0.31 ^b^	0.26 ^a^	0.27 ^a^	<0.001	0.011
Index of thrombogenicity	0.23	0.23	0.23	0.986	0.010
PUFA n-6/PUFA n-3	0.67 ^a^	0.81 ^b^	0.78 ^b^	<0.001	0.032
EPA + DHA	2.55 ^b^	1.57 ^a^	1.98 ^a,b^	<0.001	0.362
Sampling at 90 d					
Index of atherogenicity	0.32 ^c^	0.27 ^a^	0.28 ^b^	<0.001	0.010
Index of thrombogenicity	0.23	0.23	0.23	0.858	0.010
PUFA n-6/PUFA n-3	0.70 ^a^	0.79 ^b^	0.76 ^a,b^	0.02	0.045
EPA + DHA	2.64 ^b^	1.68 ^a^	2.27 ^a,b^	<0.01	0.413
	Red muscle				
Initial sampling					
Index of atherogenicity	0.29	0.26	-	0.013	0.029
Index of thrombogenicity	0.22	0.23	-	0.172	0.014
PUFA n-6/PUFA n-3	0.65	0.80	-	<0.001	0.041
EPA + DHA	2.54	1.58	-	<0.001	0.177
Sampling at 45 d					
Index of atherogenicity	0.29 ^b^	0.25 ^a^	0.25 ^a^	<0.001	0.010
Index of thrombogenicity	0.22	0.23	0.21	0.328	0.010
PUFA n-6/PUFA n-3	0.62 ^a^	0.76 ^b^	0.69 ^a,b^	<0.01	0.040
EPA + DHA	2.72 ^b^	1.69 ^a^	2.06 ^a^	<0.001	0.221
Sampling at 90 d					
Index of atherogenicity	0.30 ^b^	0.25 ^a^	0.27 ^b^	<0.001	0.014
Index of thrombogenicity	0.22	0.22	0.22	0.888	0.017
PUFA n-6/PUFA n-3	0.63 ^a^	0.74 ^b^	0.69 ^a,b^	<0.01	0.044
EPA + DHA	2.36	1.97	2.40	0.10	0.377

PUFA: polyunsaturated fatty acids, RSD: residual standard deviation, EPA + DHA: C20:5 n-3 + C22:6 n-3. FO 100: diet formulated with fish oil as lipid source. FO 0: diet in which fish oil was totally substituted by vegetable oils. The feeding plan gives the diet fed during grow-out/the diet fed during wash-out. ^a,b,c^ Means with different superscript letter statistically differ.

**Table 6:** Ingredients (g·kg^−1^ as fed) and proximate composition (%dry matter) of the experimental diets.

	Diet	
	FO 100	FO 0
Fish meal	350	350
Wheat	100	100
Wheat gluten	140	140
Defatted soybean meal	185	185
Iberian meat meal	110	110
Fish oil	95	0
Linseed oil	-	38
Sunflower oil	-	28
Palm oil	-	29
Multivitamin and minerals mix ^1^	20	20
Proximate composition		
Dry matter, %	87.4	89.1
Crude protein, % DM	58.8	58.8
Crude lipid, % DM	15.9	16.6
Ash, %DM	8.4	8.3
Gross energy, MJ·kg^−1^ DM	24.3	24.4

FO 100: Diet formulated with fish oil as lipid source; FO 0: Diet in which fish oil was totally substituted with vegetable oils. ^1^ Vitamins and mineral mixture (values are g·kg^−1^): Premix, 25; Choline chloride, 10; DL-α-tocopherol, 5; ascorbic acid, 5; (PO_4_)_2_Ca_3_, 5. Premix composition (values are IU·kg^−1^): Retinol acetate, 1,000,000; calcipherol, 500; DL-α-tocopherol, 10; menadione sodium bisulphite, 0.8; hidroclorhidrate thiamine, 2.3; riboflavin, 2.3; pyridoxine hydrochloride, 15; cyanocobalamin, 25; nicotinamide, 15; pantothenic acid, 6; folic acid, 0.65; biotin, 0.07; ascorbic acid, 75; inositol, 15; betaine, 100; polypeptides, 12.

**Table 7:** Fatty acid composition (% of total fatty acid content) of the experimental diets.

	Diet	
	FO 100	FO 0
14:0	3.28 ± 0.26	1.65 ± 0.02
16:0	18.88 ± 1.14	18.93 ± 0.81
17:0	0.53 ± 0.04	0.16 ± 0.04
18:0	5.07 ± 0.20	4.71 ± 0.05
∑ SFA	27.79 ± 1.65	25.46 ± 0.82
16:1 n-9	4.22 ± 0.64	1.82 ± 0.02
18:1 n-9	27.14 ± 0.29	32.67 ± 0.29
18:1 n-7	3.94 ± 0.55	2.44 ± 0.07
22:1 n-9	0.32 ± 0.02	0.06 ± 0.00
∑ MUFA	35.62 ± 0.40	36.99 ± 0.20
18:2 n-6	12.66 ± 0.89	14.86 ± 0.87
18:3 n-6	0.10 ± 0.004	0.09 ± 0.05
20:3 n-6	0.10 ± 0.04	0.04 ± 0.004
20:4 n-6	1.02 ± 0.17	0.35 ± 0.02
22:4 n-6	0.24 ± 0.02	0.09 ± 0.002
∑ n-6 PUFA	14.12 ± 1.00	15.43 ± 0.90
18:3 n-3	2.24 ± 0.19	14.60 ± 0.01
20:3 n-3	0.15 ± 0.01	0.06 ± 0.01
20:5 n-3	5.81 ± 1.19	2.77 ± 0.001
22:5 n-3	1.29 ± 0.15	0.42 ± 0.004
22:6 n-3	12.98 ± 2.21	4.28 ± 0.12
∑ n-3 PUFA	22.47 ± 3.04	22.13 ± 0.12
∑ PUFA	36.59 ± 2.04	37.56 ± 1.03
∑ n-6/∑ n-3	0.63 ± 0.13	0.70 ± 0.04
DHA/EPA	2.23 ± 0.08	1.54 ± 0.05

FO 100: Diet formulated with fish oil as lipid source; FO 0: Diet in which fish oil was totally substituted with vegetable oils.SFA: Saturated fatty acids; MUFA: Monounsaturated fatty acids; PUFA: Polyunsaturated fatty acids; DHA/EPA: 22:6 n-3/20:5 n-3.

## Supplementary Materials

Supplementary materials can be found at https://www.mdpi.com/1422-0067/21/14/4871/s1.

## Abbreviations

AA	Arachidonic acid
ALA	α-linolenic acid
DHA	Docosahexaenoic acid
DM	Dry matter
EFA	Essential fatty acids
EPA	Eicosapentaenoic acid
FA	Fatty acids
FO	Fish oil
IA	Index of atherogenicity
IT	Index of thrombogenicity
LA	Linoleic acid
MUFA	Monounsaturated fatty acids
PUFA	Polyunsaturated fatty acids
RSD	Residual standard deviation
SFA	Saturated fatty acids
VO	Vegetable oil
WW	Wet weight

## References

[1] Chaves-Pozo E., Abellán E., Baixauli P., Arizcun M. (2019). An overview of the reproductive cycle of cultured specimens of a potential candidate for Mediterranean aquaculture, *Umbrina cirrosa*. Aquaculture.

[2] Roo J., Hernández-Cruz C., Mesa-Rodriguez A., Fernández-Palacios H., Izquierdo M.S. (2019). Effect of increasing n-3 HUFA content in enriched Artemia on growth, survival and skeleton anomalies occurrence of greater amberjack *Seriola dumerili* larvae. Aquaculture.

[3] Sicuro B., Luzzana U. (2016). The State of *Seriola* spp. Other Than Yellowtail (*S. quinqueradiata*) Farming in the World. Rev. Fish. Sci. Aquac..

[4] Codabaccus M.B., Ng W.-K., Nichols P.D., Carter C.G. (2013). Restoration of EPA and DHA in rainbow trout (*Oncorhynchus mykiss*) using a finishing fish oil diet at two different water temperatures. Food Chem..

[5] Turchini G., Torstensen B.E., Ng W.-K. (2009). Fish oil replacement in finfish nutrition. Rev. Aquac..

[6] Fountoulaki E., Vasilaki A., Hurtado R., Grigorakis K., Karacostas I., Nengas I., Rigos G., Kotzamanis Y., Venou B., Alexis M. (2009). Fish oil substitution by vegetable oils in commercial diets for gilthead sea bream (*Sparus aurata* L.); effects on growth performance, flesh quality and fillet fatty acid profile. Aquaculture.

[7] Shepherd J., Jackson A.J. (2013). Global fishmeal and fish-oil supply: Inputs, outputs and marketsa. J. Fish Biol..

[8] Naylor R.L., Hardy R.W., Bureau D., Chiu A., Elliott M., Farrell A.P., Forster I., Gatlin D.M., Goldburg R.J., Hua K. (2009). Feeding aquaculture in an era of finite resources. Proc. Natl. Acad. Sci. USA.

[9] Monge-Ortiz R., Tomás-Vidal A., Rodriguez-Barreto D., Llorens S.M., Pérez J., Jover-Cerdá M., Lorenzo A. (2018). Replacement of fish oil with vegetable oil blends in feeds for greater amberjack (*Seriola dumerili*) juveniles: Effect on growth performance, feed efficiency, tissue fatty acid composition and flesh nutritional value. Aquac. Nutr..

[10] Izquierdo M.S., Montero D., Robaina L., Caballero M.J., Rosenlund G., Ginés R. (2005). Alterations in fillet fatty acid profile and flesh quality in gilthead seabream (*Sparus aurata*) fed vegetable oils for a long term period. Recovery of fatty acid profiles by fish oil feeding. Aquaculture.

[11] Montero D., Robaina L., Caballero M.J., Ginés R., Izquierdo M.S. (2005). Growth, feed utilization and flesh quality of European sea bass (*Dicentrarchus labrax*) fed diets containing vegetable oils: A time-course study on the effect of a re-feeding period with a 100% fish oil diet. Aquaculture.

[12] Tocher D.R. (2010). Fatty acid requirements in ontogeny of marine and freshwater fish. Aquac. Res..

[13] Mourente G., Bell J. (2006). Partial replacement of dietary fish oil with blends of vegetable oils (rapeseed, linseed and palm oils) in diets for European sea bass (*Dicentrarchus labrax* L.) over a long term growth study: Effects on muscle and liver fatty acid composition and effectiveness of a fish oil finishing diet. Comp. Biochem. Physiol. Part B Biochem. Mol. Biol..

[14] Turchini G., Mentasti T., Frøyland L., Orban E., Caprino F., Moretti V.M., Valfré F. (2003). Effects of alternative dietary lipid sources on performance, tissue chemical composition, mitochondrial fatty acid oxidation capabilities and sensory characteristics in brown trout (*Salmo trutta* L.). Aquaculture.

[15] Jobling M. (2004). Are modifications in tissue fatty acid profiles following a change in diet the result of dilution?. Aquaculture.

[16] E Connor W. (2000). Importance of n-3 fatty acids in health and disease. Am. J. Clin. Nutr..

[17] Ruxton C.H.S., Reed S.C., Simpson M.J.A., Millington K.J. (2004). The health benefits of omega-3 polyunsaturated fatty acids: A review of the evidence. J. Hum. Nutr. Diet..

[18] Seierstad S.L., Seljeflot I., Johansen O., Hansen R., Haugen M., Rosenlund G., Frøyland L., Arnesen H. (2005). Dietary intake of differently fed salmon; the influence on markers of human atherosclerosis. Eur. J. Clin. Investig..

[19] Williams C.M. (2000). Dietary fatty acids and human health. Anim. Res..

[20] Olsen S.F., Secher N.J. (2002). Low consumption of seafood in early pregnancy as a risk factor for preterm delivery: Prospective cohort study. BJM.

[21] Breslow J.L. (2006). n-3 Fatty acids and cardiovascular disease. Am. J. Clin. Nutr..

[22] Rosenberg I.H. (2002). Fish—Food to calm the heart. N. Engl. J. Med..

[23] Hu F.B., Bronner L., Willett W.C., Stampfer M.J., Rexrode K.M., Albert C.M., Hunter D., Manson J.E. (2002). Fish and omega-3 fatty acid intake and risk of coronary heart disease in women. J. Am. Med. Assoc..

[24] Robin J.H., Regost C., Arzel J.S., Kaushik S. (2003). Fatty acid profile of fish following a change in dietary fatty acid source: Model of fatty acid composition with a dilution hypothesis. Aquaculture.

[25] Turchini G., Francis D.S., De Silva S.S. (2006). Modification of tissue fatty acid composition in Murray cod (*Maccullochella peelii peelii*, Mitchell) resulting from a shift from vegetable oil diets to a fish oil diet. Aquac. Res..

[26] Glencross B., Hawkins W., Curnow J. (2003). Restoration of the fatty acid composition of red seabream (*Pagrus auratus*) using a fish oil finishing diet after grow-out on plant oil based diets. Aquac. Nutr..

[27] Reis B., Cabral E.M., Fernandes T., Castro-Cunha M., Oliveira M.B.P.P., Cunha L.M., Valente L.M. (2014). Long-term feeding of vegetable oils to Senegalese sole until market size: Effects on growth and flesh quality. Recovery of fatty acid profiles by a fish oil finishing diet. Aquaculture.

[28] Turchini G.M., Francis D., De Silva S. (2007). Finishing diets stimulate compensatory growth: Results of a study on Murray cod, *Maccullochella peelii peelii*. Aquac. Nutr..

[29] Lane R.L., Trushenski J., Kohler C.C. (2006). Modification of fillet composition and evidence of differential fatty acid turnover in sunshine bass *Morone chrysops × M. saxatilis* following change in dietary lipid source. Lipids.

[30] Thanuthong T., Francis D.S., Senadheera S., Jones P., Turchini G. (2012). Short?term food deprivation before a fish oil finishing strategy improves the deposition of n-3 LC-PUFA, but not the washing?out of C18 PUFA in rainbow trout. Aquac. Nutr..

[31] Yıldız M., Eroldoğan T.O., Ofori-Mensah S., Engin K., Baltacı M.A., Yıldız M. (2018). The effects of fish oil replacement by vegetable oils on growth performance and fatty acid profile of rainbow trout: Re-feeding with fish oil finishing diet improved the fatty acid composition. Aquaculture.

[32] Bell J.G., Henderson R.J., Tocher D.R., Sargent J.R. (2004). Replacement of dietary fish oil with increasing levels of linseed oil: Modification of flesh fatty acid compositions in Atlantic salmon (*Salmo salar*) using a fish oil finishing diet. Lipids.

[33] Bell J., McGhee F., Campbell P.J., Sargent J.R. (2003). Rapeseed oil as an alternative to marine fish oil in diets of post-smolt Atlantic salmon (*Salmo salar*): Changes in flesh fatty acid composition and effectiveness of subsequent fish oil “wash out”. Aquaculture.

[34] Bell J.G., Tocher D.R., Henderson R.J., Dick J.R., Crampton V.O. (2003). Altered Fatty Acid Compositions in Atlantic Salmon (Salmo salar) Fed Diets Containing Linseed and Rapeseed Oils Can Be Partially Restored by a Subsequent Fish Oil Finishing Diet. J. Nutr..

[35] Torstensen B.E., Froyland L., Ornsrud R., Lie O. (2004). Tailoring of a cardioprotective muscle fatty acid composition of Atlantic salmon (*Salmo salar*) fed vegetable oils. Food Chem..

[36] Carpene E., Martin B., Libera L.D. (1998). Biochemical differences in lateral muscle of wild and farmed gilthead sea bream (series *Sparus aurata* L.). Fish Physiol. Biochem..

[37] McKenzie D.J., Farrell A.P. (2011). Swimming and Other Activities—Energetics of Fish Swimming. Encyclopedia of Fish Physiology: From Genome to Environment.

[38] Teulier L., Thoral E., Queiros Q., McKenzie D.J., Roussel D., Dutto G., Gasset E., Bourjea J., Saraux C. (2019). Muscle bioenergetics of two emblematic Mediterranean fish species: Sardina pilchardus and Sparus aurata. Comp. Biochem. Physiol. Part A Mol. Integr. Physiol..

[39] Palmeri G., Turchini G., De Silva S. (2007). Lipid characterisation and distribution in the fillet of the farmed Australian native fish, Murray cod (*Maccullochella peelii peelii*). Food Chem..

[40] Calder P.C. (2012). Long-chain fatty acids and inflammation. Proc. Nutr. Soc..

[41] Leaf A. (2001). Plasma nonesterified fatty acid concentration as a risk factor for sudden cardiac death: The Paris Prospective Study. Circulation.

[42] Ghaeni M., Ghahfarokhi K.N., Zaheri L. (2013). Fatty Acids Profile, Atherogenic (IA) and Thrombogenic (IT) Health Lipid Indices in Leiognathusbindus and Upeneussulphureus. J. Mar. Sci. Res. Dev..

[43] Durmus M. (2019). Fish oil for human health: Omega-3 fatty acid profiles of marine seafood species. Food Sci. Technol..

[44] Ouraji H., Shabanpour B., Kenari A.A., Shabani A., Nezami S., Sudagar M., Faghani S. (2009). Total lipid, fatty acid composition and lipid oxidation of Indian white shrimp (Fenneropenaeus indicus) fed diets containing different lipid sources. J. Sci. Food Agric..

[45] Stancheva M., Merdzhanova A., Dobreva D.A., Makedonski L. (2014). Common carp (*Cyprinus carpio*) and European catfish (*Silurus glanis*) from Danube River as sources of fat soluble vitamins and fatty acids. Czech J. Food Sci..

[46] Martínez-Llorens S., Tomás-Vidal A., Moñino A.V., Torres M.P., Jover-Cerdá M. (2007). Effects of dietary soybean oil concentration on growth, nutrient utilization and muscle fatty acid composition of gilthead seabream (*Sparus aurata* L.). Aquac. Res..

[47] Regost C., Arzel J., Cardinal M., Rosenlund G., Kaushik S.J. (2003). Total replacement of fish oil by soybean or linseed oil with a return to fish oil in Turbot (*Psetta maxima*). Aquaculture.

[48] Kiessling K.-H., Kiessling A. (1993). Selective utilization of fatty acids in rainbow trout (*Oncorhynchus mykiss* Walbaum) red muscle mitochondria. Can. J. Zoool..

[49] Linares F., Henderson R.J. (1991). Incorporation of 14C-labelled polyunsaturated fatty acids by juvenile turbot, *Scophthalmus maximus* (L.) in vivo. J. Fish Boil..

[50] Tocher D.R., Sargent J.R. (1990). Incorporation into Phospholipid Classes and Metabolism via Desaturation and Elongation of Various14C-Labelled (n-3) and (n-6) Polyunsaturated Fatty Acids in Trout Astrocytes in Primary Culture. J. Neurochem..

[51] Kiessling A., Pickova J., Johansson L., Åsgård T., Storebakken T., Kiessling K.-H. (2001). Changes in fatty acid composition in muscle and adipose tissue of farmed rainbow trout (*Oncorhynchus mykiss*) in relation to ration and age. Food Chem..

[52] Jeong B.Y., Jeong W.G., Moon S.K., Ohshima T. (2002). Preferential accumulation of fatty acids in the testis and ovary of cultured and wild sweet smelt *Plecoglossus altivelis*. Comp. Biochem. Physiol. Part B Biochem. Mol. Biol..

[53] Izquierdo M.S., Obach A., Arantzamendi L., Montero D., Robaina L., Rosenlund G. (2003). Dietary lipid sources for seabream and seabass: Growth performance, tissue composition and flesh quality. Aquac. Nutr..

[54] Bell J.G., McEvoy J., Tocher D.R., McGhee F., Campbell P.J., Sargent J.R. (2001). Replacement of fish oil with rapeseed oil in diets of Atlantic salmon (*Salmo salar*) affects tissue lipid compositions and hepatocyte fatty acid metabolism. J. Nutr..

[55] Tinsley I.J., Krueger H.M., Saddler J.B. (1973). Fatty Acid Content of Coho Salmon, *Oncorhynchus Kisutch*—A Statistical Approach to Changes Produced by Diet. J. Fish. Res. Board Can..

[56] Madsen L., Frøyland L., Dyrøy E., Helland K., Berge R.K. (1998). Docosahexaenoic and eicosapentaenoic acids are differently metabolized in rat liver during mitochondria and peroxisome proliferation. J. Lipid Res..

[57] Benedito-Palos L., Navarro J.C., Bermejo-Nogales A., Saera-Vila A., Kaushik S., Pérez-Sánchez J. (2009). The time course of fish oil wash-out follows a simple dilution model in gilthead sea bream (*Sparus aurata* L.) fed graded levels of vegetable oils. Aquaculture.

[58] Bordignon F., Tomas-Vidal A., Trocino A., Sorribes M.C.M., Jover-Cerdá M., Martínez-Llorens S. (2020). Fatty Acid Signatures in Different Tissues of Mediterranean Yellowtail, *Seriola dumerili* (Risso, 1810), Fed Diets Containing Different Levels of Vegetable and Fish Oils. Animals.

[59] AOAC (1990). International Official Methods of Analysis of AOAC International.

[60] Brouwer E., Blaxter K.L. (1965). Report of Sub-Committee on Constants and Factors. Proceedings of the 3rd Symposium on Energy Metabolism.

[61] O’Fallon J.V., Busboom J.R., Nelson M.L., Gaskins C.T. (2007). A direct method for fatty acid methyl ester synthesis: Application to wet meat tissues, oils, and feedstuffs. J. Anim. Sci..

[62] Ulbricht T., Southgate D. (1991). Coronary heart disease: Seven dietary factors. Lancet.

[63] SAS (Statistical Analysis System Institute, Inc.) (2013). SAS/STAT(R) 9.3 User’s Guide.

