# Supplementary material for: Recovery of Fatty Acid Composition in Mediterranean Yellowtail (Seriola dumerili, Risso 1810) fed a Fish-Oil Finishing Diet
Source: Int J Mol Sci. 2020 Jul 9;21(14):4871. doi: 10.3390/ijms21144871 (PMC7402285; doi:10.3390/ijms21144871)
Supplement: Supplementary file 1 [file ijms-21-04871-s001.pdf]

Supplementary Materials

Table S1. Fatty acid (FA) composition (mg of FA/g of tissue) of white and red muscles at the beginning of the wash-out period in *S. dumerili* always fed FO 100 and FO 0 diets (12 fish per diet): effect of the grow-out diet. Values are expressed as least square (LS) means.

	White muscle				Red muscle			
	**FO 100**	**FO 0**	***p*-Value**	**RSD**	**FO 100**	**FO 0**	***p*-Value**	**RSD**
Fatty acids								
14:0	4.04	2.96	<0.001	0.475	2.69	2.07	0.044	0.615
15:0	Tr	Tr			0.22	0.05	0.035	0.166
16:0	26.62	28.66	0.176	3.111	21.42	23.71	0.092	2.795
17:0	0.71	0.47	<0.001	0.087	0.65	0.39	<0.001	0.103
18:0	10.44	12.34	0.004	1.212	9.64	11.25	<0.01	1.202
20:0	0.63	0.64	0.757	0.078	0.56	0.52	0.301	0.075
22:0	0.22	0.33	0.01	0.087	0.18	0.27	<0.001	0.044
24:0	0.14	0.17	0.063	0.027	0.13	0.16	0.063	0.030
∑ SFA[1]	42.79	45.57	0.239	4.891	35.49	38.42	0.182	4.577
14:1 n-9	0.50	0.29	<0.001	0.061	0.15	0.16	0.875	0.160
16:1 n-9	6.74	4.74	<0.001	0.744	4.84	3.38	<0.001	0.750
17:1 n-10	0.59	0.38	<0.001	0.070	0.47	0.27	<0.001	0.063
18:1 n-7	7.32	6.46	0.044	0.854	6.25	5.39	0.042	0.849
18:1 n-9	49.15	64.12	<0.001	7.757	39.96	52.30	<0.001	6.100
20:1 n-9	3.06	1.52	<0.001	0.416	2.83	1.22	<0.001	0.374
22:1 n-9	0.50	0.24	<0.001	0.109	0.53	0.21	<0.001	0.163
24:1 n-9	0.61	0.39	<0.001	0.115	0.61	0.33	<0.001	0.113
∑ MUFA	68.47	78.09	0.044	9.531	55.64	63.27	0.045	7.673
18:2 n-6	25.33	33.18	<0.001	3.670	19.25	25.99	<0.001	2.917
18:3 n-6	0.25	0.25	0.902	0.041	0.19	0.17	0.368	0.037
20:3 n-6	0.18	0.11	<0.001	0.030	0.16	0.12	0.257	0.074
20:4 n-6	1.37	0.97	<0.001	0.136	1.34	0.88	<0.001	0.090
22:4 n-6	0.69	0.35	<0.001	0.088	0.74	0.35	<0.001	0.071
∑ n-6 PUFA	27.80	34.86	0.001	3.812	21.68	27.51	<0.001	3.059
18:3n-3	7.31	21.98	<0.001	3.351	4.31	16.19	<0.001	2.099
20:3n-3	0.36	0.50	0.002	0.084	0.29	0.45	<0.001	0.070
20:5n-3	6.11	4.55	<0.001	0.689	4.82	3.52	<0.001	0.487
22:5n-3	2.99	2.11	<0.001	0.366	3.12	2.12	<0.001	0.382
22:6n-3	19.99	12.77	<0.001	2.586	20.56	12.26	<0.001	1.436
∑ n-3 PUFA	36.76	41.91	0.043	5.065	33.10	34.54	0.343	3.215
20:2	1.52	0.98	<0.001	0.181	1.19	0.75	<0.001	0.164
22:2	0.74	0.37	<0.001	0.098	0.40	0.16	<0.001	0.084
∑ PUFA	66.82	78.12	0.013	8.800	56.57	63.07	0.038	6.281
DHA/EPA	3.27	2.81	0.002	0.258	4.30	3.50	<0.001	0.344

SFA: saturated fatty acids; MUFA: monounsaturated fatty acids; PUFA: polyunsaturated fatty acids; RSD: residual standard deviation; DHA/EPA: C22:6 n-3/ C20:5 n-3. FO 100: diet formulated with fish oil as lipid source. FO 0: diet in which fish oil was totally substituted by vegetable oil. [1] Total SFA include fatty acids not listed (<0.01 mg FA/g of tissue), C6:0, C8:0, C10:0, C11:0, C12:0, C13:0, C21:0, C23:0.

Table S2. Fatty acid (FA) composition (mg of FA/g of tissue) of white and red muscle in *S. dumerili* fed FO 100/FO 100, FO 0/FO 0, and FO 0/FO 100 diets after 45 d of wash-out (6 fish per feeding plan): effect of the feeding plan. Values are expressed as least square (LS) means.

	White muscle					Red muscle				
**Feeding plan**	**FO 100/FO 100**	**FO 0/FO 0**	**FO 0/FO 100**	***p*-Value**	**RSD**	**FO 100/FO 100**	**FO 0/FO 0**	**FO 0/FO 100**	***p*-Value**	**RSD**
Fat, % WW	4.79	4.65	5.42	0.554	1.057	4.37	4.67	4.42	0.842	0.808
14:0	3.45^{ab}	2.27^{a}	2.92^{a}	0.033	0.596	2.80^{c}	1.97^{a}	2.08^{b}	0.011	0.383
15:0	0.43^{c}	0.22^{ab}	0.31^{b}	0.002	0.069	0.38^{c}	0.20^{a}	0.24^{b}	<0.001	0.038
16:0	24.00	23.04	26.16	0.547	4.038	22.24	23.26	21.79	0.764	2.897
17:0	0.64^{b}	0.36^{a}	0.48^{ab}	0.003	0.097	0.61^{b}	0.36^{a}	0.43^{a}	<0.001	0.067
18:0	10.08	10.20	11.47	0.357	1.514	10.31	11.17	10.74	0.578	1.252
20:0	0.58	0.48	0.58	0.261	0.097	0.57	0.49	0.51	0.286	0.077
24:0	0.13	0.13	0.15	0.385	0.021	0.13	0.16	0.15	0.081	0.017
∑ SFA	39.51	36.94	42.34	0.508	6.362	37.22	37.89	36.16	0.871	4.667
16:1 n-9	5.88^{b}	3.68^{a}	4.76^{ab}	0.012	0.931	4.93^{b}	3.32^{a}	3.57^{a}	0.004	0.620
17:1 n-10	0.58^{b}	0.28^{a}	0.38^{ab}	<0.001	0.078	0.51^{b}	0.28^{a}	0.34^{a}	<0.001	0.046
18:1 n-7	6.84	4.95	6.19	0.059	1.078	6.80	5.52	5.80	0.077	0.838
18:1 n-9	44.24	51.48	56.17	0.140	8.707	41.63	52.49	47.73	0.067	6.472
20:1 n-9	2.93^{b}	1.16^{a}	1.79^{a}	<0.001	0.444	2.84^{b}	1.23^{a}	1.74^{a}	<0.001	0.291
22:1 n-9	0.51^{b}	0.18^{a}	0.28^{ab}	0.002	0.111	0.53^{b}	0.22^{a}	0.33^{a}	<0.001	0.065
24:1 n-9	0.67^{b}	0.30^{a}	0.40^{a}	0.002	0.127	0.62^{b}	0.34^{a}	0.41^{a}	<0.001	0.058
∑ MUFA	61.62	62.02	69.96	0.489	11.21	57.86	63.41	59.93	0.591	8.190
18:2 n-6	21.60	25.53	28.12	0.059	3.801	19.39	24.78	22.52	0.051	3.027
18:3 n-6	0.23	0.20	0.24	0.413	0.042	0.18	0.18	0.17	0.879	0.027
20:3 n-6	0.17^{b}	0.09^{a}	0.12^{ab}	0.011	0.035	0.17^{b}	0.10^{a}	0.11^{a}	0.008	0.033
20:4 n-6	1.46^{b}	0.86^{a}	1.11^{a}	<0.001	0.146	1.45^{c}	0.96^{a}	1.16^{b}	<0.001	0.091
22:4 n-6	0.77^{b}	0.29^{a}	0.46^{a}	<0.001	0.084	0.80^{c}	0.36^{a}	0.51^{b}	<0.001	0.050
∑ n-6 PUFA	24.22	26.97	30.03	0.125	4.018	22.00	26.38	24.47	0.139	3.169
18:3 n-3	5.50^{a}	16.62^{b}	16.20^{b}	<0.001	2.832	4.66^{a}	15.12^{b}	11.54^{b}	<0.001	2.125
20:3 n-3	0.33	0.41	0.46	0.099	0.090	0.31^{a}	0.47^{b}	0.48^{b}	0.025	0.093
20:5 n-3	5.67^{b}	3.50^{a}	4.50^{ab}	0.012	0.921	4.82^{b}	3.50^{a}	3.90^{ab}	0.017	0.617
22:5 n-3	2.95^{b}	1.76^{a}	2.27^{a}	<0.001	0.350	3.37^{b}	2.42^{a}	2.71^{a}	0.004	0.359
22:6 n-3	21.38^{b}	11.11^{a}	15.27^{a}	<0.001	2.202	22.30^{b}	13.39^{a}	16.72^{ab}	<0.001	1.686
∑ n-3 PUFA	35.83	33.40	38.71	0.377	5.140	35.46	34.90	35.35	0.978	4.178
20:2	1.37^{b}	0.77^{a}	1.02^{ab}	0.005	0.228	1.21^{b}	0.77^{a}	0.89^{a}	0.003	0.157
22:2	0.69^{b}	0.28^{a}	0.42^{a}	<0.001	0.116	0.60^{b}	0.27^{a}	0.37^{a}	<0.001	0.072
∑ PUFA	62.11	61.42	70.17	0.358	9.392	59.27	62.37	61.08	0.811	7.402
DHA/EPA	3.83	3.23	3.42	0.13	0.438	4.69^{b}	3.86^{a}	4.28^{ab}	0.04	0.428

SFA: saturated fatty acids, MUFA: monounsaturated fatty acids, PUFA: polyunsaturated fatty acids, RSD: residual standard deviation, DHA/EPA: C22:6 n-3/ C20:5 n-3. FO 100: diet formulated with fish oil as lipid source. FO 0: diet in which fish oil was totally substituted by vegetable oil. The feeding plan gives the diet fed during grow-out /the diet fed during wash-out. ^{a,b,c} Means with different superscript letter statistically differ. [1] Total SFA include fatty acids not listed (<0.01 mg FA/g of tissue), C6:0, C8:0, C10:0, C11:0, C12:0, C13:0, C21:0, C23:0.

Table S3. Fatty acid (FA) composition (mg of FA/g of tissue) of white and red muscle in *S. dumerili* fed FO 100/ FO 100, FO 0/ FO 0, and FO 0/ FO 100 diets after 90 d of wash-out (6 fish per feeding plan): effect of the feeding plan. Values are expressed as least square (LS) means.

	White muscle					Red muscle				
**Feeding plan**	**FO 100/FO 100**	**FO 0/FO 0**	**FO 0/FO 100**	***p*-Value**	**RSD**	**FO 100/FO 100**	**FO 0/FO 0**	**FO 0/FO 100**	***p*-Value**	**RSD**
Fatty acids										
14:0	3.87	2.91	3.49	0.242	0.970	2.60	2.43	2.76	0.735	0.710
15:0	0.50^{b}	0.28^{a}	0.40^{ab}	0.016	0.117	0.37	0.25	0.67	0.107	0.315
16:0	25.14	28.68	28.90	0.513	5.734	19.78^{a}	27.93^{b}	25.79^{ab}	0.017	4.658
17:0	0.70^{b}	0.45^{a}	0.59^{ab}	0.026	0.141	0.57	0.46	0.56	0.272	0.134
18:0	10.34	12.29	12.03	0.268	2.068	9.21^{a}	13.28^{b}	11.68^{ab}	0.006	1.977
20:0	0.60	0.58	0.66	0.654	0.142	0.51	0.64	0.59	0.168	0.122
22:0	0.18^{a}	0.30^{b}	0.28^{ab}	0.010	0.056	0.17^{a}	0.33^{b}	0.26^{a}	<0.001	0.047
24:0	0.12	0.15	0.16	0.205	0.032	0.12^{a}	0.19^{b}	0.19^{b}	0.037	0.044
∑ SFA[1]	41.45	45.65	46.50	0.658	9.193	33.33^{a}	45.50^{b}	42.54^{ab}	0.031	7.686
16:1 n-9	6.48	4.67	5.71	0.158	1.572	4.42	4.06	4.68	0.667	1.161
17:1 n-10	0.56^{b}	0.32^{a}	0.47^{ab}	0.009	0.119	0.47	0. 34	0.44	0.154	0.120
18:1 n-7	7.12	5.97	7.35	0.388	1.823	5.86	6.56	6.92	0.465	1.374
18:1 n-9	46.61	65.99	61.80	0.056	12.90	35.87^{a}	65.35^{b}	55.45^{a}	<0.001	9.702
20:1 n-9	3.18^{b}	1.39^{a}	2.33^{ab}	0.003	0.746	2.58^{b}	1.49^{a}	2.27^{ab}	0.012	0.600
22:1 n-9	0.57^{b}	0.18^{a}	0.40^{b}	<0.001	0.132	0.51^{b}	0.28^{a}	0.42^{ab}	0.006	0.114
24:1 n-9	0.70^{b}	0.35^{a}	0.52^{ab}	<0.001	0.119	0.67^{b}	0.46^{a}	0.54^{ab}	0.048	0.142
∑ MUFA	65.21	78.86	78.57	0.358	17.12	50.38^{a}	78.55^{b}	70.73^{ab}	0.004	13.07
18:2 n-6	22.84	30.68	28.97	0.100	5.967	16.76^{a}	29.47^{c}	25.20^{b}	<0.001	4.247
18:3 n-6	0.21	0.24	0.23	0.824	0.073	0.17	0.22	0.26	0.253	0.077
20:3 n-6	0.39	0.14	0.27	0.365	0.305	0.15	0.13	0.15	0.418	0.032
20:4 n-6	1.54^{b}	0.96^{a}	1.26^{ab}	0.003	0.238	1.31^{b}	1.06^{a}	1.32^{b}	0.037	0.185
22:4 n-6	0.66^{b}	0.39^{a}	0.54^{ab}	0.035	0.161	0.65^{b}	0.41^{a}	0.57^{ab}	0.033	0.155
∑ n-6 PUFA	25.63	32.40	31.27	0.215	6.553	19.04^{a}	31.31^{b}	27.50^{a}	<0.001	4.605
18:3 n-3	4.59^{a}	21.64^{c}	15.40^{b}	<0.001	2.809	3.09^{a}	19.03^{c}	12.44^{b}	<0.001	1.680
20:3 n-3	0.31^{a}	0.57^{b}	0.49^{ab}	0.006	0.117	0.25^{a}	0.65^{b}	0.48^{a}	<0.001	0.114
20:5 n-3	5.87	4.42	5.15	0.175	1.282	4.07	4.54	4.53	0.697	1.096
22:5 n-3	3.16^{b}	2.07^{a}	2.55^{ab}	0.031	0.636	2.86	2.81	2.99	0.894	0.609
22:6 n-3	21.84^{b}	12.25^{a}	17.51^{b}	<0.001	2.894	19.53^{b}	15.11^{a}	19.48^{ab}	0.022	2.933
∑ n-3 PUFA	35.76	40.96	41.09	0.384	6.872	29.79^{a}	42.15^{b}	39.92^{a}	0.003	5.645
20:2	1.42	0.98	1.24	0.098	0.333	1.04	0.97	1.11	0.734	0.278
22:2	0.73^{b}	0.37^{a}	0.54^{ab}	0.010	0.179	0.53	0.36	0.55	0.094	0.164
∑ PUFA	63.54	74.71	74.15	0.358	13.89	50.40^{a}	74.78^{b}	69.06^{a}	0.002	10.58
DHA/EPA	3.86^{b}	2.78^{a}	3.44^{b}	<0.001	0.297	5.10^{b}	3.41^{a}	4.33^{ab}	<0.01	0.714

SFA: saturated fatty acids, MUFA: monounsaturated fatty acids, PUFA: polyunsaturated fatty acids, RSD: residual standard deviation, DHA/EPA: C22:6 n-3/ C20:5 n-3. FO 100: diet formulated with fish oil as lipid source. FO 0: diet in which fish oil was totally substituted by vegetable oil. The feeding plan gives the diet fed during grow-out /the diet fed during wash-out. ^{a,b,c} Means with different superscript letter statistically differ. [1]Total SFA include fatty acids not listed (<0.01 mg FA/g of tissue), C6:0, C8:0, C10:0, C11:0, C12:0, C13:0, C21:0, C23:0.
